# Implementation of an Emergency Medicine Research Associates Program: Sharing 20 Years of Experience

**DOI:** 10.5811/westjem.2017.12.36721

**Published:** 2018-03-08

**Authors:** Beau Abar, Vincent DeRienzo, Joseph Glick, Nancy Wood, Manish N. Shah, Sandra Schneider, David Adler

**Affiliations:** * University of Rochester Medical Center, Department of Emergency Medicine, Rochester, New York; † University of Wisconsin-Madison, School of Medicine and Public Health, Department of Emergency Medicine, Madison, Wisconsin; ‡ Hofstra University, Hofstra Northwell School of Medicine, Department of Emergency Medicine, Hempstead, New York; § American College of Emergency Physicians, Irving Texas

## Abstract

**Introduction:**

The use of research associates (RA) programs to facilitate study enrollment in the emergency department was initiated during the mid-1990s. The University of Rochester Medical Center (URMC) was an early adopting site for this model, which has experienced considerable growth and development over the past 20 years.

**Methods:**

Our goal was to detail the Emergency Department Research Associates (EDRA) program processes developed at the URMC that has led to our program’s sustainability and productivity. These processes, and the lessons learned during their development, can assist institutions seeking to establish an RA program or refine an existing program.

**Results:**

Defined procedures for selecting, training, and monitoring EDRAs have been created and refined with the goal of maximizing study enrollment and minimizing protocol deviations. Our EDRA program functions as a paid service center for investigators, and our EDRAs engage in a variety of study-related activities including screening and enrolling patients, administering surveys, collecting bio-specimens, and making follow-up calls. Over the past two years, our program has averaged 222 enrollments/month (standard deviation = 79.93), gathering roughly 25 participants per study per month.

**Conclusion:**

Our EDRA model has consistently resulted in some of the highest number of enrollments across a variety of recently funded, multi-center studies. Maintaining a high-quality EDRA program requires continual investment on the part of the leadership team, though the benefits to investigators within and outside the department outweigh these costs.

## INTRODUCTION

Research in emergency medicine has been accelerating rapidly over the past several decades, with increasing quality and quantity of publications and grant mechanisms. Concomitant to the increased output have been maturations in processes and procedures for conducting widely heterogeneous research in the emergency department (ED) setting. In the fast-paced ED environment, practicing clinicians have historically found it difficult to identify and enroll patients into their research studies.^1^ One particular innovation that has been adopted and refined with considerable success is the use of a research associates (RA) program for study enrollment and procedures in the ED.^2^–^5^

In the mid-1990s, Drs. Judd Hollander,^6^ Keith Bradley,^2^ and others pioneered the use of undergraduate, pre-health profession students to enroll subjects into investigator-initiated research studies and perform basic study procedures. This early work has led to the development of numerous RA programs across the United States and significant expansion of the scope of research performed in emergency medicine. The Department of Emergency Medicine at the University of Rochester Medical Center (URMC) was a very early adopter of this model.^7^ Our 20-year-old RA program has experienced considerable development and expansion since its inception. In the past 10 years alone, over 20,000 study participants have been enrolled by the URMC Emergency Department RA (EDRA) program into a wide variety of research studies. The EDRA program has been responsible for URMC being among the top enrolling institutions in the majority of the recent multi-center ED studies in which we participate.

For example, in the past year our center (a) was the second among 22 participating centers to meet the enrollment goal in a National Cancer Institute-supported study of ED utilization by patients with active cancer, despite being late to join the consortium; (b) was the highest enrolling site among 11 sites, enrolling over 1,000 subjects (accounting for 30% of total enrollment), into a National Heart, Lung, & Blood Institute-funded multi-center study of syncope in older adults; and (c) was the top enrolling site in several industry-sponsored, multisite clinical trials and validation studies on mild traumatic brain injury. Recent research using our program has been published in outlets including *Academic Emergency Medicine, Journal of Emergency Medicine, Annals of Emergency Medicine, Western Journal of Emergency Medicine, the American Journal of Emergency Medicine, PLOS One, Prehospital Emergency Care, Pediatric Emergency Care, Prehospital and Disaster Medicine, Journal of Trauma, JAMA Oncology, Journal of the American Geriatric Society,* and *Psychiatric Services*. Given this success, our team is frequently asked to share our model with collaborators across the country. Our goal here was to describe the evolution of our RA program model from its early roots, present quantitative evidence of our program activities, and provide a brief overview of our structure and processes for investigators interested in program creation, refinement, and/or expansion.

## THE EDRA PROGRAM AT THE UNIVERSITY OF ROCHESTER

Our EDRA program aims to maximize recruitment for research studies within the department. Each faculty member in the research division of our ED has published using EDRA-collected data. Furthermore, more clinically focused EM faculty have also frequently published using our EDRA program, as the program strengths in data acquisition complement the clinical knowledge of these providers. Our program has also established a significant institutional profile, as investigators from numerous other departments and divisions frequently use our EDRA program to recruit patients into their studies. For example, in the past six months alone, our program has worked with the departments/divisions of infectious disease, laboratory medicine, obstetrics, psychiatry, and pediatrics, among others. For context, Strong Memorial Hospital (SMH), where the vast majority of our EDRA-related research takes place, is a Level I ACS trauma center, regional stroke center, regional heart center, and burn center with 830 beds. The ED in which the program is housed employs 85 fulltime faculty members and 42 residents (14 per class; three-year program). The SMH ED, including the pediatric ED, is a 29,000 square-foot unit that in 2016 saw over 116,000 patients. The University of Rochester, directly next door to SMH, had an enrollment of 6,170 fulltime undergraduate students during the 2016–2017 academic year.

**Figure 1:**
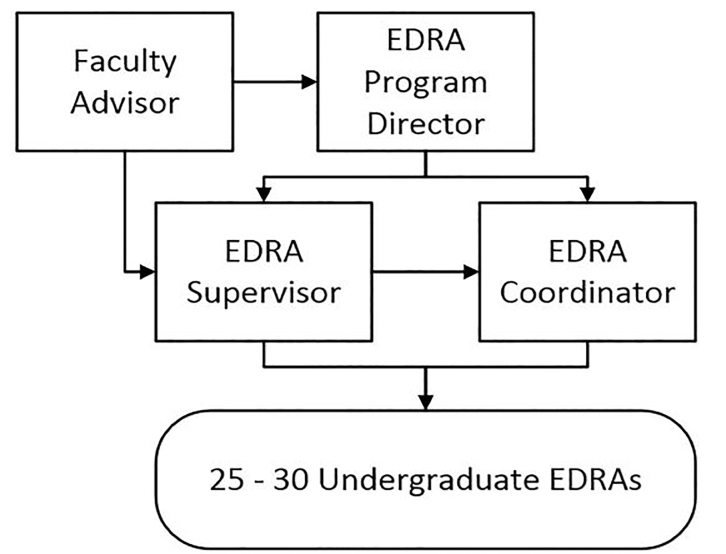
Emergency Department Research Associates (EDRA) program personnel structure.

Over the past 20 years,^7^ the procedures and infrastructure governing our EDRA program have been formalized and refined to enhance its effectiveness and efficiency. The program is currently structured with (a) a faculty advisor who provides scientific oversight of proposed research and long-term direction (5% salary support provided); (b) a program director who interacts with study teams interested in using the EDRA program and oversees the EDRA coordinator and supervisor (25% effort); (c) a full-time post-baccalaureate supervisor who is responsible for hiring and training EDRAs, scheduling coverage of the ED by EDRAs, generating quotes for EDRA program usage by study teams, and formalizing and implementing study-specific protocols; (d) a half-time college senior/post-baccalaureate coordinator responsible for assisting the EDRA supervisor and piloting study protocols in the ED; and (e) 18–35 hourly-paid undergraduate EDRAs (see Figure 1). Over the past two years (including summer months), we have averaged 30.79 EDRAs on the payroll per month (standard deviation [SD] = 4.51).

Our EDRA program is structured as a university service center, with funding received from investigators using our services and as-needed support from the ED. (Institutional funding is eliminated when the program is fully funded by investigators). The need for departmental support is mitigated through monthly budget reconciliation meetings where program costs and revenues are discussed and managed to avoid propagation of a deficit. Program costs consist primarily of faculty/administrative effort, EDRA salaries (students are paid an hourly wage), and training expenses (including hourly EDRA costs, administrative fees, and phlebotomy course registrations), resulting in a fixed hourly rate for program utilization. This hourly rate is adjusted annually with the goal of cost neutrality in accordance with cost center status.

Investigators interested in using our program contact our team, meet with the program director, supervisor, and coordinator to discuss their project and potential levels of EDRA involvement, and then work with our team on planning the operationalization of their protocol. Pertinent information for this process includes the length of the study, the amount of initial and continuing study-specific training required, the number of patients expected to be screened (determined by eligibility criteria and estimates following electronic medical record [EMR] data abstraction performed by the EDRA staff), expected patient enrollment, and duration of EDRA study procedures (in minutes) including screening, consent, enrollment, and/or follow-up. This phase is particularly important for investigators from other departments as they often require guidance regarding the clinical workflow of the ED and our developed best practices for facilitating enrollment and study success.

Investigators then work with our team to develop an acceptable quote for service based on our annually fixed program rate and the agreed-upon amount of time EDRAs would devote to the specific project. EDRA time includes administrative startup time and continual program oversight, EDRA training, and EDRA coverage of the ED for 16 hours per day, seven days per week, with 8–12 of those hours necessitating two EDRAs to keep up with the patient volume and study demands. The total investigator cost for using our program is highly variable, from short-term studies with simple screening and referral requirements that cost as little as $1,000 to larger studies with more extensive EDRA involvement entailing program budgets in excess of $65,000 over the course of four years. The EDRA budget for any given study is modified, as needed, as the project progresses.

Importantly, our EDRA leadership team is closely linked with our departmental research review committee (chaired by the EDRA faculty advisor), which evaluates research protocols involving the ED for potential human subjects, clinical flow, and scientific concerns before investigators can receive institutional review board approval. This involvement allows the EDRA program to assist investigators in the creation of effective, efficient, and ethical research protocols. Given our track record of successful completion of enrollment protocols, human subjects training, and leadership oversight, our program has applied for and received “umbrella approval” from the URMC review board for EDRA involvement in enrollment, consent, and basic study procedures (e.g., survey administration, interviews, nasal swabs) across different research studies. When an investigator references this annually reviewed umbrella protocol, it allows our team of EDRAs to participate in disparate research projects without the potentially burdensome paperwork associated with including each EDRA as study personnel on each project.

## HIRING AND TRAINING EDRAS

Our program compensates the EDRAs both experientially and monetarily, as students are hired as part-time staff. While other models for RA programs have demonstrated considerable success^3^,^5^,^7^,^8^, we have found the paid-enroller model to be most successful at our institution with regard to the quality of student applicants and retention of employees.

The University of Rochester has an undergraduate population that is very enthusiastic about careers in health sciences given the proximity and accessibility of the URMC (campuses directly adjacent). This close proximity to an exceptional undergraduate population is, no doubt, a strong asset to our program and significantly contributes to our program’s success (though high-quality RA programs in settings with much looser connection to an undergraduate population have demonstrated considerable success^8^). Although there is no set criteria for who will be successful in this position, there are a few key indicators that help identify potentially successful candidates from the perennially large applicant pool, including grade point average, academic major, research experience at the college level, professional and/or volunteer experience working with people, favorable recommendations from previous employers, and communication skills demonstrated during the interviews for the position.

Given that the EDRA position is paid and has demonstrated significant student post-graduate success, we are fortunate to receive a competitive pool of applicants during each hiring cycle (i.e., approximately 15–20 applicants per position). It is not only our reputation and compensation that ensures our high quality EDRA team. We ask our senior EDRAs for recommendations of peers who they think would be successful in the position, send solicitations via e-mail to apply for an EDRA position to pre-medical and health-science student groups, give announcements to traditional pre-medical curriculum classes when the application periods are open, and attend Student Employment Office (SEO) events such as the bi-annual undergraduate job fair.

Once applications have been submitted to the SEO, additional paper applications are sent to all interested applicants that solicit short answers regarding previous work, clinical experiences, and research involvement. From the paper applications, roughly 25% of these applicants are granted half-hour interviews to further evaluate their ability and experience, of whom roughly one-third are accepted. The total number of EDRA position offers made during each hiring cycle is based on position needs. Over the past two years, we have averaged seven new EDRAs across five hiring classes. The majority of our accepted EDRAs are in their second or third year of undergraduate education. This ensures that our EDRAs are committed to their future in healthcare and have had sufficient time to acclimate to the college atmosphere, as well as reduces the amount of new hires that leave the position before providing the program with at least a year of service. As a result, the average length of time EDRAs have served in the program, based on completed terms of employment since Fall 2013, is 20.0 months (SD = 8.90).

**Figure 2:**
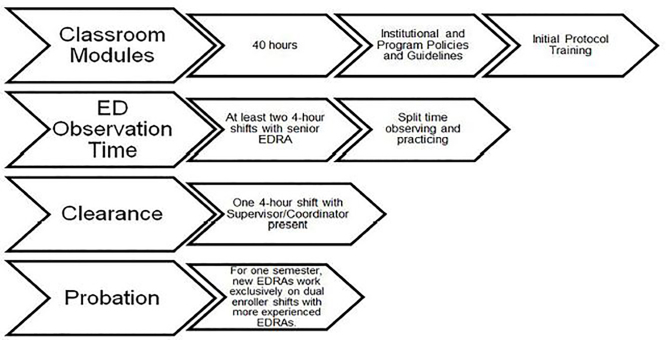
Emergency Department Research Associates (EDRA) initial training timeline.

Training for each EDRA is extensive and consistent (Figure 2). Initial training consists of 40 hours of classroom time and 12 hours of supervised clinical time before they are cleared to work independently in the ED. The classroom learning topics include EDRA and URMC policies and guidelines, university-sanctioned Health Insurance Portability and Accountability Act training (including Collaborative Institutional Training Initiative certification), the informed consent process (both practical application and historical context), study-specific trainings, professional interaction development, survey administration, and standardized good-documentation practices. Once the classroom portion of the initial training is complete, EDRAs are given a two-hour, in-depth guided tour of the ED. The new EDRAs then work two four-hour “shadow shifts” where they observe the on-shift behavior of a senior EDRA. The new hires then work one complete four-hour shift while the program coordinator observes them and evaluates their readiness to function independently based on their (a) facility with our EMR; (b) ability to appropriately consent patients; (c) knowledge of and aptitude in study procedures; (d) communication timing and quality with clinical providers on shift; and (e) navigation of the ED.

Due to the longevity of EDRAs in the program and the length of certain studies, the initial training is insufficient to maintain a high quality of work. EDRAs receive weekly update notifications via e-mail. Monthly staff meetings are held to communicate major updates and protocol changes, as well as providing EDRAs an opportunity to discuss any obstacles they may have encountered. This opportunity for peer interaction and problem solving afforded by these meetings has proven to be a very effective means of developing and communicating best practices relative to specific trials. When new studies are accepted, EDRAs are called in for new study-specific trainings.

For more involved protocols, in-service “boot camp” training sessions are held with the entire EDRA staff, including a review of all study documents, procedures, and best practices in patient approach. These in-service trainings occur 1–2 months after the initiation of the protocol and again every six months for the duration of the protocol. A similar investment in continuing education is the quality assurance process that the program undertakes. All errors in screening, enrollment, or other study-related procedures and guidelines are addressed individually within one week. This time-sensitive response allows for the mistake to be promptly corrected and for the EDRA to learn from the mistake.

Because the EDRAs are college students, there are often long periods of excused absence while they are with the program (e.g., summer recess, semester abroad). During these absences, other EDRAs increase their hours spent enrolling (e.g., a subset of EDRAs work ≥ 30 hours/week during the summer) or additional EDRAs are hired to sustain the quality of service provided by our program. Those EDRAs who enroll on a more intensive schedule during the winter and/or summer breaks tend to develop rapidly, as they are afforded greater opportunities to refine their patient-approach style. To maintain the quality of the work upon an EDRA’s return from an absence, EDRAs have three learning modules to complete: an online policy review with a competency check; a classroom review of study and training updates; and an abbreviated evaluation shift with the EDRA coordinator.

In addition to our extensive training protocols, we have developed a rigorous quality assurance (QA) process for monitoring study progress and efficiency, as well as for providing quantifiable, formative feedback to the EDRAs. Specifically, EDRAs are required to maintain a shift chart including the name, medical record number, chief complaint, and research disposition of all eligible patients arriving to the ED during enrollment hours. Twice a month, every visit to the ED is pulled in an EMR report for age, sex, chief complaint, method of arrival, and diagnosis. These data are then compared to the hard-copy shift charts that the EDRAs use to ensure that all potentially eligible patients are being screened during enrollment hours. The shift chart allows the EDRA to demonstrate that work is being done, even on patients who are not eligible, who refuse to participate, or who are missed for other reasons.

Formal performance assessments are done quarterly throughout the program, as well as at the end of the EDRA’s time as a student or at the request of the individual. These assessments are based on feedback from clinical and research staff, and focus on five core competencies identified in successful EDRAs: communication skills, community interaction skills, critical thinking, personal presentation, and policy adherence. EDRAs are also assessed on their enrollment performance and any quality assurance and improvement concerns that have arisen. These assessments also serve as a mechanism by which EDRA readiness can be evaluated for additional clinical and research opportunities.

## TYPICAL EDRA RESEARCH ACTIVITIES

Monitoring of patients presenting to the ED is required for nearly all of the studies that our EDRA program engages in. As representatives of the Department of EmergencyMedicine, our EDRAs are able to use the URMC EMR track-board to monitor the basic characteristics of patients presenting to the ED. Each EDRA has a separate EMR login with rights to view information on ED patients, and each login is monitored by hospital administration for appropriate usage (e.g., only viewing study-related information; limiting access to sensitive behavioral/psychiatric health information). EDRAs use this tool to initially screen patients for eligibility (e.g., age range, presenting complaint, method of arrival). Some studies then require the EDRA to contact a principal investigator or study coordinator to alert them of a potentially eligible patient, at which point our program involvement with the patient may be complete. Other studies require the EDRA to approach those patients who meet the inclusion criteria in the EMR. EDRAs then introduce themselves and the study to the patient, offer to answer any questions, determine capacity for providing consent using standardized procedures appropriate for the particular study,^9^ and obtain and document informed consent (whether verbal or written).

**Figure 3:**
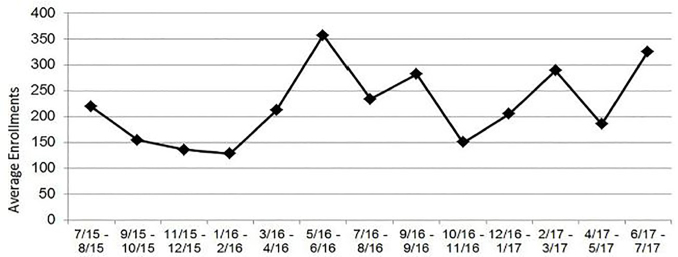
Emergency Department Research Associates monthly enrollments over time.

Over the past 26 months, EDRAs have averaged 222 enrolled participants per month (SD = 79.92; see Figure 3). This average actually underrepresents EDRA recruitment activities, as it does not include enrollments in studies where EDRAs are only responsible for identifying potential participants and notifying study teams. During this same time period, our program has been actively enrolling for an average of 8.98 studies at a time (SD = 1.61; range = 7 – 12). Importantly, across the wide variety of studies our EDRAs enroll in, we have demonstrated a monthly average rate of 2.99 enrollments for every patient refusal (SD = 1.10).

The responsibilities of our EDRAs following consent are highly variable. Certain research protocols require the EDRA to contact the contracted study team to hand off the consented patient, while others require the EDRA to perform study procedures. These procedures could include administering surveys, obtaining specimens (e.g., nasal swabs, saliva, blood), or performing brief interventions (e.g., brief motivational interviewing, referral to treatment).

Our EDRAs are also often required to approach and survey providers (e.g., emergency physicians, nurses, emergency medical technicians) involved in the care of enrolled patients. We have developed procedures for collecting needed study information without hampering clinical efforts, and clinical leadership provides consistent support for our research efforts.

## OPPORTUNITIES FOR EXCEPTIONAL EDRAS

Our program at URMC has developed a system for both rewarding EDRAs who demonstrate excellence and facilitating sustainability/institutional memory long term. Specifically, the breadth and depth of studies that our program is simultaneously engaged in often requires additional performance analysis, data management, and administrative work. Those EDRAs who perform well enrolling patients and who demonstrate an interest in expanding their experience level are used to fill these roles on a temporary basis (e.g., 2–3 weeks to three months). Developing this experience base greatly assists our long-term efforts for the program, as we primarily hire into the EDRA supervisor and coordinator positions from within the program.

Our program is also relatively unique among RA programs with regard to clinical integration. Specifically, since the summer of 2010 senior members of the EDRA program who show significant aptitude have been offered positions as a provider assistant and liaison (PAL). The PAL position provides an undergraduate support staff person to attending emergency physicians in the ED during peak hours to improve provider productivity, residency teaching and tracking, and patient satisfaction. PALs work side by side with attending physicians to help maintain assessments details (e.g., expedite delivery of lab and imaging results when needed), make phone calls (e.g., contacting primary care physicians and hospital consults), coordinate discharge resources, care for patient comfort needs, assist with traumas and critically ill patients in a non-medical role, and assist visitors. We have received consistent feedback from providers that the PAL program helps improve ED efficiency. Over the past five years, PALs have provided an average of 142.18 hours of service in the ED per month (SD = 48.56).

We also offer exceptional EDRAs study-specific training opportunities that will generalize to their future work. For example, a subset of our EDRA team recently received phlebotomy training and certification to better facilitate enrollment on several of our ongoing studies. Other EDRAs have become certified to work in a clinical laboratory as a way to enhance the efficiency of a set of studies examining biomarkers among ED patients. These students are then able to perform study-related procedures outside of the ED. Our program aims to continually develop these types of skills among our EDRAs to enhance our capacity for engaging in a broad set of research protocols.

## CONCLUSION

The EDRA program at URMC has evolved significantly over its 20 years of service to the university. Our current model has consistently resulted in high enrollment rates across a variety of recently funded, multi-center studies. Much of this success can be attributed to (a) the formalized, extensive, and continuous training of the EDRAs, and (b) program efforts to integrate EDRAs into the clinical environment (i.e., dedicated space in the ED, access to electronic medical records). Nascent programs are encouraged to place much of their efforts into these specific areas of program development, as well as identifying and formalizing methods for accessing high-quality, engaged enrollers (e.g., undergraduate institutions, non-profit organizations, etc.). The design of our program has allowed for continual improvement in the quality of the enroller workforce, with the majority of students applying for and enrolling in post-graduate education in the health professions. This improvement and expansion in the URMC EDRA program requires continual investment on the part of the leadership team and ED, as a whole, though the benefits to investigators within and outside the department significantly outweigh these costs.
